# The mediating effect of post-traumatic growth on the relationship between personality traits and resilience among a sample of Lebanese adults

**DOI:** 10.1371/journal.pone.0298043

**Published:** 2024-05-17

**Authors:** Zeinab Bitar, Feten Fekih-Romdhane, Daniella Mahfoud, Mirna Fawaz, Souheil Hallit, Sahar Obeid

**Affiliations:** 1 Univ Rennes, Inserm, EHESP, Irset (Institut de recherche en santé, environnement et travail), Rennes, UMR_S 1085, F-35000, France; 2 Department of Psychiatry “Ibn Omrane”, The Tunisian Center of Early Intervention in Psychosis, Razi hospital, Manouba, Tunisia; 3 Faculty of Medicine of Tunis, Tunis El Manar University, Tunis, Tunisia; 4 Department of Ophthalmology, Eye N’ Brain Research Group, Yong Loo Lin School of Medicine, National University of Singapore, Singapore, Singapore; 5 College of Health Sciences, American University of the Middle East, Kuwait, Kuwait; 6 School of Medicine and Medical Sciences, Holy Spirit University of Kaslik, Jounieh, Lebanon; 7 Applied Science Research Center, Applied Science Private University, Amman, Jordan; 8 Social and Education Sciences Department, School of Arts and Sciences, Lebanese American University, Jbeil, Lebanon; Qatar University College of Nursing, QATAR

## Abstract

**Background:**

Resilience plays a crucial role in mental health promotion and prevention, and was shown to be more represented in individuals who exhibit high levels of extraversion, openness, agreeableness, and conscientiousness. However, there is a lack of studies that comprehensively investigate the association between personality traits and resilience in Lebanon and Arab countries more broadly. The purpose of the present study was to complement the literature by investigating the direct and indirect effects between the five personality traits and resilience among a sample of Lebanese adults through the intermediary role of posttraumatic growth.

**Methodology:**

A cross-sectional study was carried out between May and July 2022, and enrolled 387 participants, all aged above 18 years old and recruited from all Lebanon governorates. The questionnaire used included socio-demographic questions, and the following scales: Connor-Davidson Resilience Scale (CD-RISC) to assess resilience, post traumatic growth (PTG), and Big Five Inventory (BFI-2). The SPSS software v.25 was used for the statistical analysis.

**Results:**

Post-traumatic growth mediated the association between extraversion / agreeableness / conscientiousness and resilience. Higher extraversion / agreeableness / conscientiousness was significantly associated with more post-traumatic growth. Higher post-traumatic growth was significantly associated with more resilience. Extraversion, but not agreeableness and conscientiousness, was significantly and directly associated with more resilience.

**Conclusion:**

Findings suggest that fostering PTG in individuals who experience adversity can help promote their resilience. Hence, it could be beneficial to design and apply programs aiming at supporting PTG among people who experience stressful and traumatizing situations, to consequently help them increase their sense of resilience.

## Background

The increased prevalence of traumatic and tragic events, such as life changes, illness, pandemics, natural disasters, or wars, has generated significant interest from the fields of neuroscience, mental health, and psychology regarding their impact on mental well-being [1]. Such stressful experiences have detrimental effects on mental health (e.g., [2–4]). However, not all individuals will be affected to the same extent. Many individuals exposed to trauma experience no significant negative psychological consequences or recover quickly from adversity [5,6]. One reason for variations in response to trauma and a key factor in achieving good mental health is resilience [6]. Some researchers define resilience as the ability to respond positively to stressful situations [7], while others view it as the ability to function normally facing persistent stress [8,9]. Resilience is defined by the American Psychological Society as the act of "bouncing back" from tough events and effectively adjusting in the face of adversity, trauma, tragedy, threats, or severe levels of stress [10]. When encountering difficulties, challenges and frustrations, individuals with higher resilience levels are often able to rapidly adapt the balance between situational needs and behavioral responses [11,12]. Resilience can, therefore, be regarded as a major personal asset which may itself be affected by several factors such as personality traits [13].

### The relationship between personality traits and resilience

Personality traits refer to unique characteristic patterns that derive and control each individual’s thoughts, feelings and behaviors in a stable and sustained way influence on individual behavior and thoughts [14]. The widely used five-factor model (FFM) [15] categorizes personality traits as extraversion (sociability and optimism), agreeableness (understanding and empathy), conscientiousness (organization, responsibility, and punctuality), openness (acceptance of new ideas and creativity), and neuroticism (mood instability and negative emotions) [16]. Previous studies found moderate association between resilience and FFM traits, with estimated average correlation coefficients for resilience ranging from −0.46 for neuroticism to 0.42 for extraversion and conscientiousness [17]. Resilient individuals tend to have high levels of openness, conscientiousness, agreeableness, and extraversion, and low levels of neuroticism [18]. Earlier research suggested that extravert individuals who face stressful situations tend to exhibit more adaptive stress management [19]. Generally, there is strong evidence that resilience is inversely associated with neuroticism and positively related to other personality traits (for meta-analysis, see [17]). In particular, extraversion and neuroticism traits were found to be significantly more impacted by life events [20,21], and to be, in turn, more specifically linked to resilience [22]. Considering that resilience could potentially be the key to explaining how individuals exposed to stressful experiences deal with adversity across the lifespan and since the ways individuals respond to adversity stem from personality traits among other factors [17], it is of high importance to investigate the mechanisms underlying the relationship between the big Five personality traits and resilience. This study proposed to complement the existing literature by investigating the role of a potential mediator in this relationship, namely post-traumatic growth (PTG).

### PTG as mediator

Posttraumatic growth (PTG) refers to a positive psychological change and cognitive adaptation following trauma, arising from surviving the experience and perceiving new life opportunities [23]. Tedeschi and Calhoun [24] distinguished between resilience and PTG by highlighting that development after trauma involves transformative cognitive rebuilding. This process may be reflected through having an increased sense of personal strength, relating to others more, realizing new possibilities in life, gaining a greater appreciation for life, and making a spiritual or existential change [24]. PTG entails a movement beyond pre-trauma levels of adaptation, whereas resilience means moving forward after difficult situations. As both PTG and resilience refer to dynamic psychological processes and transformation occurring after a trauma, PTG is assumed to have a close connection with resilience [25,26]. From a theoretical perspective, positive psychological change experienced after potentially challenging circumstances could predict resilience. Indeed, PTG implies enhancement of the self and becoming stronger in the aftermath of trauma, which increases capacity for action and resistance to detrimental impacts [27,28]. Previous studies demonstrated that PTG is closely associated with resilience [25,26]. In other words, it has been suggested that experiencing PTG after trauma is related to an increased likelihood of exhibiting resilience characteristics [29–31]. On the other hand, agreeableness, neuroticism, and extraversion were demonstrated to be significantly linked to posttraumatic growth (PTG), with agreeableness and extraversion positively affecting PTG and neuroticism having a negative impact [32]. Previous research also suggested a positive association between openness to experience, extraversion, and PTG, with these personality traits reflecting a greater inclination for flexibility, exploration, and adaptation [33].

### The present study

Before the 2020 pandemic, Lebanese faced crises in sanitation management, disruptions in essential services like water and electricity, as well as Lebanese pound corruption, impacting mental health and resilience [34]. Following the Beirut blast, the remarkable resilience and adaptability of the Lebanese population in the face of trauma, tragedy, threats, or any significant source of stress became evident in their daily lives [35]. Based on the literature above, the present study aims to test the following hypotheses: (1) the big five personality traits of non-clinical Lebanese adults may positively affect their resilience levels; (2) the big five personality traits may indirectly affect resilience through the intermediary role of PTG.

## Methods

### Study design

A cross-sectional study conducted between May and July 2022, enrolled 387 participants through convenience sampling in several Lebanese governorates (Beirut, Mount Lebanon, North Lebanon, South Lebanon, and Bekaa). The consent form, study purpose, anonymity assurance, and questionnaire were all sent to participants via an internet survey link. The link was distributed over social media using a snowball approach. All participants above the age of 18 were eligible to participate, and those who refused to complete the questionnaire were excluded.

### Ethics approval and consent to participate

This study protocol (HPC-023-2022) was approved by the Psychiatric Hospital of the Cross Ethics and Research Committee. When submitting the online form, each participant was required to provide written informed consent. All procedures were carried out in conformity with the relevant guidelines and regulations.

### Minimal sample size calculation

According to the G-power software [36], a minimum of 316 students was deemed necessary to have enough statistical power, based on a 5% risk of error, 80% power, f2 = 2.5% and 10 factors to be entered in the multivariable analysis.

### Questionnaire

The questionnaire used was anonymous and in Arabic, the native language in Lebanon; it required approximately 10 to 15 minutes to complete. It consisted of two parts. The first part gathered participants’ sociodemographic information including age, gender, region of living, marital status and education level. The Household Crowding Index (HCI), a measure of family’s socioeconomic status [37], was calculated by dividing the number of individuals living in the house by the number of rooms (excluding the kitchen and the bathrooms). The scales used in the present study were included in the second part.

#### Connor-Davidson Resilience Scale (CD-RISC)

The CD-RISC contains ten items [38,39] each of which are scored on a 5-point scale ranging from 0 (*not true at all*) to 4 (*true nearly all of the time*); for example, “I am able to adapt when changes occur” and “I am not easily discouraged by failure.” Higher scores on the CD-RISC-10 indicate higher levels of resilience (Cronbach’s alpha in this study = .88). The Arabic validated version of the CD-RISC was used [40].

#### Post traumatic growth (PTG)

This scale is composed of 10 items and measures favorable outcomes after a traumatic event [41]. PTG items are rated between 0 to 5 with 0 indicates “I didn’t experience this change as a result of my crisis” and 5 indicated “I experienced this change to a very great degree as a result of my crisis” [41]. Higher score means higher post traumatic growth (Cronbach’s alpha in this study = .95). This scale is validated in Arabic [42].

#### Big Five Inventory (BFI-2)

The questionnaire consists of 60 items assessing various personality traits (48). Items are scored on a 5-point Likert scale ranging from strongly disagree (1 point) to strongly agree (5 points). Higher scores would indicate higher level of the personality trait. The Arabic BFI-2 was sent to us by Dr Soto [43], and previously used in Lebanese populations (e.g., [44]). The Cronbach’s alpha values in this study were as follows: extroversion (.70), agreeableness (.76), conscientiousness (.72), negative emotionality (.77) and open mindedness (.74).

### Statistical analysis

The SPSS software v.25 has been used for the statistical analysis. The resilience and PTG scores were considered normally distributed since the skewness and kurtosis values varied between -1 and +1 [45]. The Student-t test was used to compare two means and the Pearson test was used to correlate two continuous variables. The mediation analysis was conducted using PROCESS MACRO (an SPSS add-on) v.3.4 model 4; four pathways derived from this analysis: pathway A from the independent variable to the mediator, pathway B from the mediator to the dependent variable, Pathways C and C’ indicating the total and direct effects from the independent to the dependent variable. Results adjusted over age, gender, marital status, education level and household crowding index and the other four personality traits. We considered the mediation analysis to be significant if the Boot Confidence Interval did not pass by zero. P<0.05 was deemed statistically significant.

## Results

### Sociodemographic and other characteristics of the sample

Three hundred eighty-seven participants participated in this study, with a mean age of 26.17 ± 11.47 years and 58.4% females. Other descriptive statistics of the sample can be found in Table 1.

**Table 1:** Sociodemographic and other characteristics of the sample (N = 387).

Variable	N (%)
Sex	
Male	161 (41.6%)
Female	226 (58.4%)
Marital status	
Single	311 (80.4%)
Married	76 (19.6%)
Education level	
Secondary or less	66 (17.1%)
University	321 (82.9%)
Region of living	
Urban	294 (76.0%)
Rural	93 (24.0%)
	**Mean ± SD**
Age (years)	26.17 ± 11.47
Household crowding index (persons/room)	1.47 ± 1.00
Resilience	23.88 ± 7.29
Post-traumatic growth	28.04 ± 11.78
Depression	4.50 ± 2.51
Anxiety	4.38 ± 2.62
Stress	3.34 ± 1.67
Extroversion	38.91 ± 5.45
Agreeableness	41.81 ± 6.12
Conscientiousness	42.26 ± 7.31
Negative emotionality	36.09 ± 6.02
Open mindedness	39.43 ± 5.24

### Bivariate analysis of factors associated with resilience

The results of the bivariate analysis of factors associated with resilience are summarized in Tables 2 and 3. The results showed that extroversion, agreeableness, conscientiousness and open mindedness were significantly associated with more resilience and post-traumatic growth, whereas negative emotionality was significantly associated with less resilience.

**Table 2:** Bivariate analysis of factors associated with resilience.

Variable	Resilience score (mean ± SD)	*t*	*df*	*p*
Sex		1.52	385	.131
Male	24.54 ± 7.69			
Female	23.40 ± 6.98			
Marital status		1.61	385	.108
Single	24.17 ± 7.34			
Married	22.67 ± 7.04			
Education level		-1.52	385	.130
Secondary or less	22.64 ± 7.09			
University	24.13 ± 7.32			
Region of living		-1.04	385	.301
Urban	23.66 ± 6.98			
Rural	24.56 ± 8.20			

**Table 3:** Correlations of continuous variables with resilience.

	1	2	3	4	5	6	7	8	9
1. Resilience	1								
2. Post-traumatic growth	.47***	1							
3. Extroversion	.34***	.33***	1						
4. Agreeableness	.26***	.35***	.47***	1					
5. Conscientiousness	.30***	.37***	.49***	.64***	1				
6. Negative emotionality	-.30***	-.09	-.19***	-.12*	-.10	1			
7. Open mindedness	.22***	.27***	.47***	.46***	.55***	.05	1		
8. Age	-.07	-.01	.03	-.01	.07	.03	-.03	1	
9. Household crowding index	-.09	.003	.03	.13*	.10	.02	.06	.13*	1

*p < .05

**p < .01

***p < .001.

### Mediation analysis

**Fig 1:**
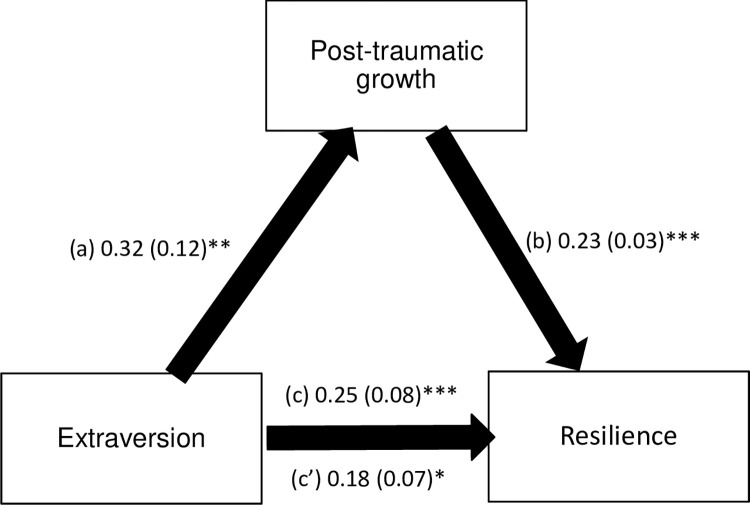
(a) Relation between extraversion and post-traumatic growth (R^2^ = .191); (b) Relation between post-traumatic growth and resilience (R^2^ = .332); (c) Total effect of extraversion on resilience (R^2^ = .222); (c’) Direct effect of extraversion on resilience. Numbers are displayed as regression coefficient (standard error). *p < 0.05; **p < 0.01; ***p < 0.001.

**Fig 2:**
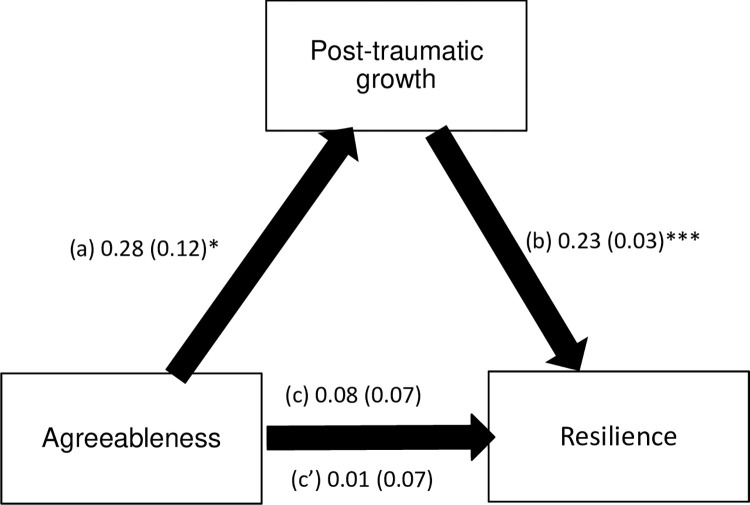
(a) Relation between agreebleness and post-traumatic growth (R^2^ = .191); (b) Relation between post-traumatic growth and resilience (R^2^ = .332); (c) Total effect of agreebleness on resilience (R^2^ = .222); (c’) Direct effect of agreebleness on resilience. Numbers are displayed as regression coefficient (standard error). *p < 0.05; ***p < 0.001.

**Fig 3:**
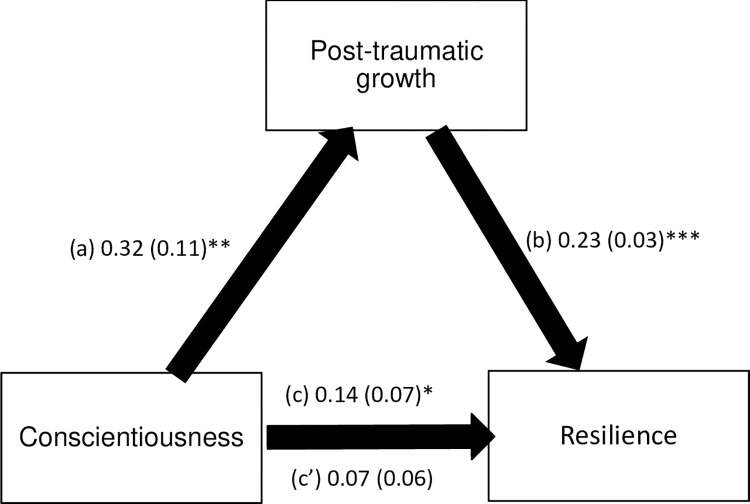
(a) Relation between conscientiousness and post-traumatic growth (R^2^ = .191); (b) Relation between post-traumatic growth and resilience (R^2^ = .332); (c) Total effect of conscientiousness on resilience (R^2^ = .222); (c’) Direct effect of conscientiousness on resilience. Numbers are displayed as regression coefficient (standard error). *p < 0.05; **p < 0.01; ***p < 0.001.

PTG mediated the association between extraversion / agreeableness / conscientiousness traits and resilience (Table 4). Higher extraversion / agreeableness / conscientiousness was significantly associated with more PTG. Higher PTG was significantly associated with more resilience. Extraversion, but not agreeableness and conscientiousness, was significantly and directly associated with more resilience (Figs 1–3).

**Table 4:** Mediation analyses results, taking each personality trait as an independent variable, post-traumatic growth as the mediator and resilience as the dependent variable.

	Direct effect			Indirect effect		
Independent variable	Beta	SE	*P*	Beta	Boot SE	Boot CI
Extraversion	.18	.07	.012	.07	.03	.01; .14*
Agreeableness	.01	.07	.842	.06	.03	.002; .14*
Conscientiousness	.07	.06	.283	.07	.03	.02; .13*
Negative emotionality	-.27	.05	< .001	-.01	.02	-.06; .04
Open mindedness	.06	.08	.439	.02	.03	-.04; .07

*indicates significant mediation. Direct effect refers to the direct association between each personality trait and resilience without the effect of the mediator, whereas the indirect effect refers to the same association through the mediator (post-traumatic growth).

## Discussion

Resilience plays a crucial role in mental health promotion and prevention [46], and was shown to be more represented in individuals who exhibit high levels of extraversion, openness, agreeableness, and conscientiousness[17]. However, there is a lack of studies that comprehensively investigate the association between personality traits and resilience in Lebanon and Arab countries more broadly. The purpose of the present study was to investigate the direct and indirect effects between the five personality traits and resilience through the intermediary role of PTG.

As expected, findings revealed that more extroverted, conscientious, and agreeable individuals reported significantly increased resilience in our study. This is in line with previous findings that PTG significantly affects resilience [47]. This emphasized the importance of developing a mature and adaptable personality is advantageous for effectively coping with and recovering from stressful situations, as resilience involves adapting to life challenges and traumatic events. In addition, these findings increase awareness of persons’ personality, which help people to understand their strengths and weakness in coping with life’s challenges, and predict one’s negative and positive aspects of wellbeing; especially among Lebanese adults imposed to burdens.

Additionally, analyses showed that PTG acted either as a partial or as a full mediator in the personality-resilience association.

Consistent with our findings, prior studies showed a positive correlation between conscientiousness, extroversion and agreeableness with each dimension of the resilience, as these traits aid in handling challenges, enhancing adaptability, and fostering positive interpersonal interactions [16,48]. Notably, it would be difficult to compare our findings with previous literature data, as research examining the mediating effect of PTG in the relationship between personality traits and resilience was not available. This study’s results suggest that, by means of high levels of PTG, individuals with certain stable personality traits [49] may activate beneficial coping strategies [50]; for example, conscientious people seek distractions or take action to resolve problems. Another explanation could be that high levels of extroversion, conscientiousness, agreeableness and high levels of PTG are linked to a strong sense of having control over circumstances, which may increase the ability to act proactively and resist to potential negative outcomes [27,28].

### Study limitations and strengths

Limitations of our study include the cross-sectional design, hindering causal inference. In addition, participants may have over or under estimated some questions, which may result in information bias. A web-based method was adopted to collect data, which might have limited the generalizability of our sample to the broader general adult population, especially since this approach has mostly attracted young adult participants. Finally, residual confounding bias is possible since other variables influencing resilience were not considered in this study. However, our study was the first to assess the mediating effect of PTG on the association between personality and resilience using validated scales and sound sample size.

### Clinical implications

The current study provides further support to the positive association between specific personality traits (i.e., extraversion, agreeableness, conscientiousness) and resilience. Findings showed that this might occur through both direct and indirect effects. Highly extroverted, conscientious, and agreeable individuals tend to report greater levels of PTG, which subsequently contribute to experiencing a heightened sense of resilience. This suggests that fostering PTG in individuals who experience adversity can help promote their resilience. This means that it could be beneficial to design and apply programs aiming at supporting PTG among people who experience stressful and traumatizing situations, to consequently help them increase their sense of resilience. Future research is still required to identify other potential mechanisms (mediators/moderators) underscoring the relation personality-resilience.

## Conclusion

In light of the present findings, it can be suggested that developing the main personality traits contributing to resilience in the Lebanese population fosters a better adapted and resilient personality for coping with daily stressors and crises. The mediating effect of PTG between personality traits and resilience implies that there is a strong need to develop and implement intervention strategies targeting PTG, as this might potentially enhance resilience in individuals who face adversity. Future longitudinal studies are needed to confirm our assumptions and deepen knowledge on the mechanisms affecting the relationship between personality and resilience.

## References

[1] Southwick SM, Bonanno GA, Masten AS, Panter-Brick C, Yehuda R. Resilience definitions, theory, and challenges: interdisciplinary perspectives. Eur J Psychotraumatol. 2014 Oct 1;5: doi: 10.3402/ejpt.v5.25338 25317257 PMC4185134

[2] Bryant RA, Schnurr PP, Pedlar D, 5-Eyes Mental Health Research and Innovation Collaboration in military and veteran mental health. Addressing the mental health needs of civilian combatants in Ukraine. Lancet Psychiatry. 2022 May;9(5):346–7. 35305300 10.1016/S2215-0366(22)00097-9

[3] Fekih-Romdhane F, Ghrissi F, Abbassi B, Cherif W, Cheour M. Prevalence and predictors of PTSD during the COVID-19 pandemic: Findings from a Tunisian community sample. Psychiatry Res. 2020 Aug;290:113131. 32485488 10.1016/j.psychres.2020.113131 PMC7255192

[4] El Zouki CJ, Chahine A, Mhanna M, Obeid S, Hallit S. Rate and correlates of post-traumatic stress disorder (PTSD) following the Beirut blast and the economic crisis among Lebanese University students: a cross-sectional study. BMC Psychiatry. 2022 Aug 5;22(1):532. doi: 10.1186/s12888-022-04180-y 35931970 PMC9356397

[5] Bonanno GA, Westphal M, Mancini AD. Resilience to loss and potential trauma. Annu Rev Clin Psychol. 2011;7:511–35. doi: 10.1146/annurev-clinpsy-032210-104526 21091190

[6] Kalisch R, Baker DG, Basten U, Boks MP, Bonanno GA, Brummelman E, et al. The resilience framework as a strategy to combat stress-related disorders. Nat Hum Behav. 2017 Nov;1(11):784–90. doi: 10.1038/s41562-017-0200-8 31024125

[7] Luthar SS, Cicchetti D, Becker B. The construct of resilience: a critical evaluation and guidelines for future work. Child Dev. 2000 Jun;71(3):543–62. doi: 10.1111/1467-8624.00164 10953923 PMC1885202

[8] Mortazavi N, Yarollahi NA. Meta-analysis of the relationship between resilience and mental health. 2015 Jun 1;

[9] Bonanno GA. Loss, Trauma, and Human Resilience: Have We Underestimated the Human Capacity to Thrive After Extremely Aversive Events? American Psychologist. 2004;59(1):20–8. doi: 10.1037/0003-066X.59.1.20 14736317

[10] American Psychological Association. Resilience [Internet]. https://www.apa.org. 2022 [cited 2022 Aug 11]. Available from: https://www.apa.org/topics/resilience.

[11] Masten AS. Resilience in children threatened by extreme adversity: Frameworks for research, practice, and translational synergy. Dev Psychopathol. 2011 May;23(2):493–506. doi: 10.1017/S0954579411000198 23786691

[12] Zimmerman MA, Stoddard SA, Eisman AB, Caldwell CH, Aiyer SM, Miller A. Adolescent Resilience: Promotive Factors That Inform Prevention. Child Dev Perspect. 2013 Dec 1;7(4). doi: 10.1111/cdep.12042 24288578 PMC3839856

[13] Alonso-Tapia J, Rodríguez-Rey R, Garrido-Hernansaiz H, Ruiz M, Nieto C. Coping, personality and resilience: Prediction of subjective resilience from coping strategies and protective personality factors. Behavioral Psychology. 2019;27(3):375–89.

[14] Satchell L, Hoskins S, Corr P, Moore R. Ruminating on the nature of intelligence: Personality predicts implicit theories and educational persistence. Personality and Individual Differences. 2017 Jul;113:109–14.

[15] Distel MA, Trull TJ, Willemsen G, Vink JM, Derom CA, Lynskey M, Martin NG, et al. The five-factor model of personality and borderline personality disorder: a genetic analysis of comorbidity. Biol Psychiatry. 2009 Dec 15;66(12):1131–8. doi: 10.1016/j.biopsych.2009.07.017 19748081

[16] Coco M, Guerrera CS, Santisi G, Riggio F, Grasso R, Di Corrado D, et al. Psychosocial Impact and Role of Resilience on Healthcare Workers during COVID-19 Pandemic. Sustainability. 2021 Jan;13(13):7096.

[17] Oshio A, Taku K, Hirano M, Saeed G. Resilience and Big Five personality traits: A meta-analysis. Personality and Individual Differences. 2018 Jun;127:54–60.

[18] Alessandri G, Vecchione M, Donnellan BM, Eisenberg N, Caprara GV, Cieciuch J. On the cross-cultural replicability of the resilient, undercontrolled, and overcontrolled personality types. J Pers. 2014 Aug;82(4):340–53. doi: 10.1111/jopy.12065 23957593

[19] Schneider TR, Rench TA, Lyons JB, Riffle RR. The influence of neuroticism, extraversion and openness on stress responses. Stress Health. 2012 Apr;28(2):102–10. doi: 10.1002/smi.1409 22281953

[20] Ogle CM, Rubin DC, Siegler IC. Changes in neuroticism following trauma exposure. J Pers. 2014 Apr;82(2):93–102. doi: 10.1111/jopy.12037 23550961 PMC3926905

[21] Sarubin N, Wolf M, Giegling I, Hilbert S, Naumann F, Gutt D, et al. Neuroticism and extraversion as mediators between positive/negative life events and resilience. Personality and Individual Differences. 2015;82:193–8.

[22] Lü W, Wang Z, Liu Y, Zhang H. Resilience as a mediator between extraversion, neuroticism and happiness, PA and NA. Personality and Individual Differences. 2014 Jun 1;63:128–33.

[23] Wagner AC, Torbit L, Jenzer T, Landy MSH, Pukay-Martin ND, Macdonald A, et al. The Role of Posttraumatic Growth in a Randomized Controlled Trial of Cognitive-Behavioral Conjoint Therapy for PTSD. J Trauma Stress. 2016 Aug;29(4):379–83. doi: 10.1002/jts.22122 27434598 PMC4988872

[24] Tedeschi RG, Calhoun LG. The Posttraumatic Growth Inventory: measuring the positive legacy of trauma. J Trauma Stress. 1996 Jul;9(3):455–71. doi: 10.1007/BF02103658 8827649

[25] Henson C, Truchot D, Canevello A. Factors that hinder post-traumatic growth: A systematic review. Encephale. 2022 Oct;48(5):560–2. doi: 10.1016/j.encep.2022.02.001 35725520

[26] Li L, Hou Y, Kang F, Wei X. The mediating and moderating roles of resilience in the relationship between anxiety, depression, and post-traumatic growth among breast cancer patients based on structural equation modeling: An observational study. Medicine. 2020 Dec 11;99(50):e23273. doi: 10.1097/MD.0000000000023273 33327251 PMC7738130

[27] Tedeschi RG, Calhoun LG. Posttraumatic Growth: Conceptual Foundations and Empirical Evidence. Psychological Inquiry. 2004;15(1):1–18.

[28] Zeng W, Wu X, Xu Y, Wu J, Zeng Y, Shao J, et al. The Impact of General Self-Efficacy on Psychological Resilience During the COVID-19 Pandemic: The Mediating Role of Posttraumatic Growth and the Moderating Role of Deliberate Rumination. Front Psychol. 2021;12:684354. doi: 10.3389/fpsyg.2021.684354 34248788 PMC8261126

[29] Yu Y, Peng L, Chen L, Long L, He W, Li M, Wang T. Resilience and social support promote posttraumatic growth of women with infertility: the mediating role of positive coping. Psychiatry Res. 2014 Feb 28;215(2):401–5. doi: 10.1016/j.psychres.2013.10.032 24368061

[30] Duan W, Guo P, Gan P. Relationships among Trait Resilience, Virtues, Post-traumatic Stress Disorder, and Post-traumatic Growth. Kavushansky A, editor. PLoS ONE. 2015 May 1;10(5):e0125707. doi: 10.1371/journal.pone.0125707 25932954 PMC4416702

[31] Bensimon M. Elaboration on the association between trauma, PTSD and posttraumatic growth: The role of trait resilience. Personality and Individual Differences. 2012 May;52(7):782–7.

[32] Mi Young K, Yujeong K. Factors Affecting Posttraumatic Growth Among College Students. TONURSJ. 2018 Dec 21;12(1):238–47.

[33] Jayawickreme E, Infurna FJ, Alajak K, Blackie LER, Chopik WJ, Chung JM, et al. Post-Traumatic Growth as Positive Personality Change: Challenges, Opportunities and Recommendations. J Pers. 2021 Feb;89(1):145–65. doi: 10.1111/jopy.12591 32897574 PMC8062071

[34] Nawyn S, Karaoğlu E, Gasteyer S, Mansour R, Ghassani A, Marquart-Pyatt S. Resilience to Nested Crises: The Effects of the Beirut Explosion on COVID-19 Safety Protocol Adherence During Humanitarian Assistance to Refugees. Front Public Health. 2022 Jul 5;10:870158. doi: 10.3389/fpubh.2022.870158 35865250 PMC9294164

[35] Abed AE, Razzak RA, Hashim HT. Mental Health Effects of COVID-19 Within the Socioeconomic Crisis and After the Beirut Blast Among Health Care Workers and Medical Students in Lebanon. Prim Care Companion CNS Disord. 2021 Jul 15;23(4):21m02977. doi: 10.4088/PCC.21m02977 34265874

[36] Faul F, Erdfelder E, Lang AG, Buchner A. G*Power 3: A flexible statistical power analysis program for the social, behavioral, and biomedical sciences. Behavior Research Methods. 2007 May;39(2):175–91. doi: 10.3758/bf03193146 17695343

[37] Melki IS. Household crowding index: a correlate of socioeconomic status and inter-pregnancy spacing in an urban setting. Journal of Epidemiology & Community Health. 2004 Jun 1;58(6):476–80. 15143115 10.1136/jech.2003.012690 PMC1732777

[38] Campbell-Sills L, Stein MB. Psychometric analysis and refinement of the connor–davidson resilience scale (CD-RISC): Validation of a 10-item measure of resilience. J Traum Stress. 2007 Dec;20(6):1019–28. doi: 10.1002/jts.20271 18157881

[39] Connor KM, Davidson JRT. Development of a new resilience scale: The Connor-Davidson Resilience Scale (CD-RISC). Depress Anxiety. 2003 Sep;18(2):76–82. doi: 10.1002/da.10113 12964174

[40] Fekih-Romdhane F, Fawaz M, Hallit R, Sawma T, Obeid S, Hallit S. Psychometric properties of an Arabic translation of the multidimensional social support scale (MSPSS) in a community sample of adults. BMC Psychiatry. 2023 Jun 14;23(1):432. doi: 10.1186/s12888-023-04937-z 37316897 PMC10265861

[41] Cann A, Calhoun LG, Tedeschi RG, Taku K, Vishnevsky T, Triplett KN, et al. A short form of the Posttraumatic Growth Inventory. Anxiety, Stress & Coping. 2010 Mar;23(2):127–37. 19582640 10.1080/10615800903094273

[42] Fekih-Romdhane F, Fawaz M, Hallit R, Sawma T, Hallit R, Hallit S. Psychometric Properties of an Arabic Translation of the 10-item Connor-Davidson Resilience scale (CD-RISC-10), the 8- and 10-item Post-Traumatic Growth Inventory-Short Form (PTGI-SF) scales [Internet]. In Review; 2023 Mar [cited 2023 Nov 16]. Available from: https://www.researchsquare.com/article/rs-2708480/v1. 10.1371/journal.pone.0293079 PMC10760825 38166051

[43] Alansari B. The Big Five Inventory (BFI): Reliability and validity of its Arabic translation in non clinical sample. Eur psychiatr. 2016 Mar;33(S1):S209–10.

[44] Moussa S, Achkouty I, Malaeb D, Ghosn A, Obeid S, Hallit S. Personality traits and perceived cognitive function in lebanese healthcare professionals. BMC Psychol. 2023 Mar 31;11(1):90. doi: 10.1186/s40359-023-01139-w 37004098 PMC10063952

[45] Hair JF, editor. A primer on partial least squares structural equation modeling (PLS-SEM). Second edition. Los Angeles: Sage; 2017. 363 p.

[46] Färber F, Rosendahl J. Trait resilience and mental health in older adults: A meta-analytic review. Personal Ment Health. 2020 Nov;14(4):361–75. doi: 10.1002/pmh.1490 32573068

[47] Yi SJ, Kim KS, Lee S, Lee H. Effects of Post Traumatic Growth on Successful Aging in Breast Cancer Survivors in South Korea: The Mediating Effect of Resilience and Intolerance of Uncertainty. Healthcare. 2023 Oct 28;11(21):2843. doi: 10.3390/healthcare11212843 37957988 PMC10650018

[48] Deng Q, Zheng B, Chen J. The Relationship Between Personality Traits, Resilience, School Support, and Creative Teaching in Higher School Physical Education Teachers. Frontiers in Psychology [Internet]. 2020 [cited 2022 Aug 22];11. Available from: https://www.frontiersin.org/articles/10.3389/fpsyg.2020.568906 33071897 10.3389/fpsyg.2020.568906 PMC7541699

[49] Harris MA, Brett CE, Johnson W, Deary IJ. Personality stability from age 14 to age 77 years. Psychology and Aging. 2016 Dec;31(8):862–74. doi: 10.1037/pag0000133 27929341 PMC5144810

[50] Hawkley LC, Cacioppo JT. Social connectedness and health. In: Human bonding: The science of affectional ties. New York, NY, US: The Guilford Press; 2013. p. 343–64.

